# Supplementary material for: Splice-Junction-Based Mapping of Alternative Isoforms in the Human Proteome
Source: Cell Rep. Author manuscript; Available in PMC 2020 Jan 15. (PMC6961840; doi:10.1016/j.celrep.2019.11.026)
Supplement: 3 [file NIHMS1546469-supplement-3.zip › DF2/PXD006675/RightAtrium-250-O14639-HFHRPDQGINIYR.pdf]

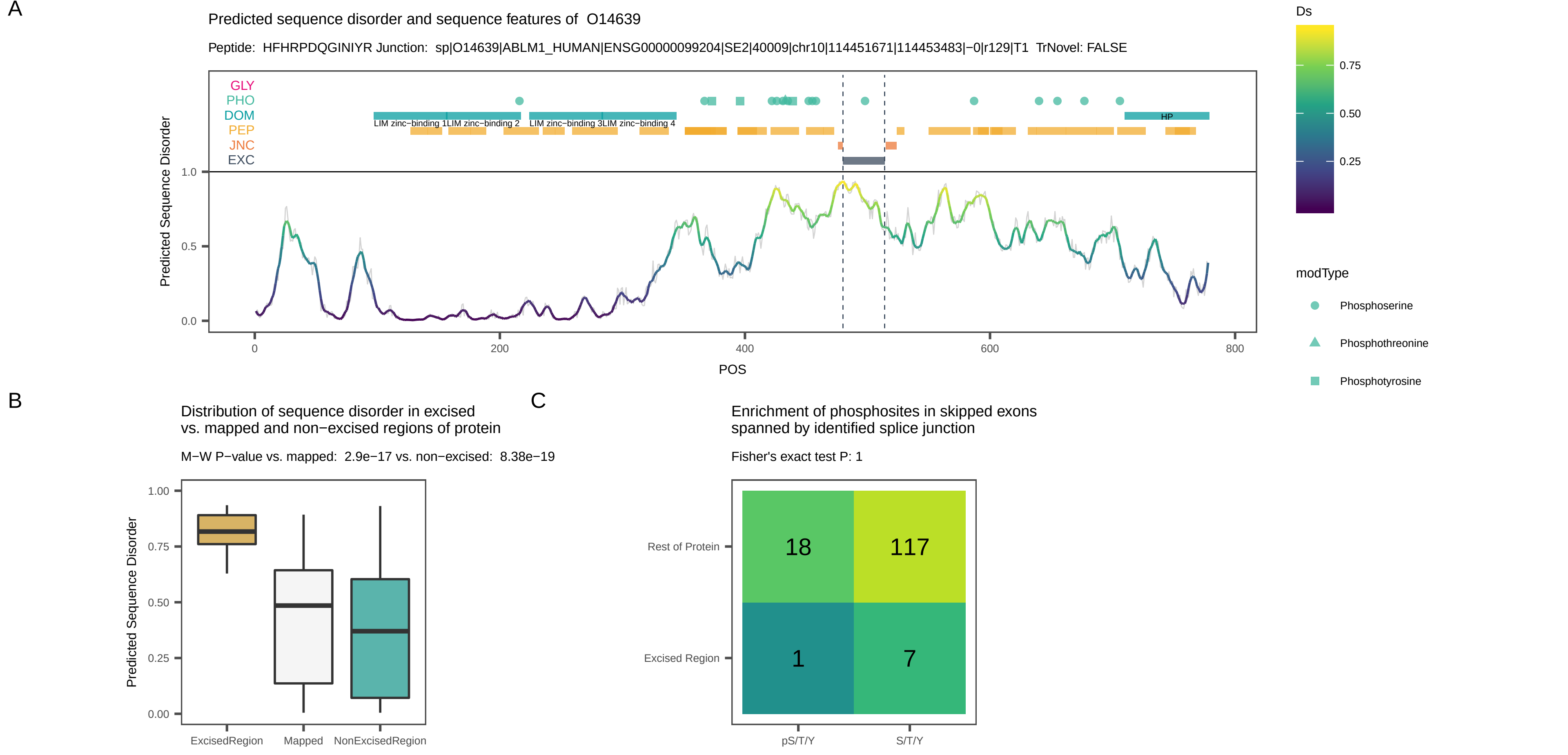
A
Predicted sequence disorder and sequence features of O14639
Peptide: HFHRPDQGINIYR Junction: sp|O14639|ABLM1_HUMAN|ENSG00000099204|SE2|40009|chr10|114451671|114453483|−0|r129|T1 TrNovel: FALSE
GLY
PHO
DOM
PEP
JNC
EXC
LIM zinc−binding 1
LIM zinc−binding 2
LIM zinc−binding 3
LIM zinc−binding 4
HP
Predicted Sequence Disorder
1.0
0.5
0.0
0
200
400
600
800
POS
Ds
0.75
0.50
0.25
modType
Phosphoserine
Phosphothreonine
Phosphotyrosine
B
Distribution of sequence disorder in excised vs. mapped and non−excised regions of protein
M−W P−value vs. mapped: 2.9e−17 vs. non−excised: 8.38e−19
Predicted Sequence Disorder
1.00
0.75
0.50
0.25
0.00
ExcisedRegion
Mapped
NonExcisedRegion
C
Enrichment of phosphosites in skipped exons spanned by identified splice junction
Fisher's exact test P: 1
Rest of Protein
Excised Region
18
117
1
7
pS/T/Y
S/T/Y
